# Quantitative physics–physiology relationship modeling of human emotional response to Shu music

**DOI:** 10.3389/fpsyg.2024.1351058

**Published:** 2024-10-08

**Authors:** Jun Su, Peng Zhou

**Affiliations:** ^1^College of Music, Chengdu Normal University, Chengdu, China; ^2^Center for Informational Biology, Key Laboratory for NeuroInformation of Ministry of Education, School of Life Science and Technology, University of Electronic Science and Technology of China, Chengdu, China

**Keywords:** quantitative physics–physiology relationship, emotional response, machine learning, random forest, Shu music

## Abstract

Music perception is one of the most complex human neurophysiological phenomena invoked by sensory stimuli, which infers an internal representation of the structured events present in a piece of music and then forms long-term echoic memory for the music. An intrinsic relationship between the basic acoustic property (physics) of music and human emotional response (physiology) to the music is suggested, which can be statistically modeled and explained by using a novel notion termed as quantitative physics–physiology relationship (QPPR). Here, we systematically analyzed the complex response profile of people to traditional/ancient music in the Shu area, a geographical concept located in the Southwest China and one of three major origins of the Chinese nation. Chill was utilized as an indicator to characterize the response strength of 18 subjects to an in-house compiled repertoire of 86 music samples, consequently creating a systematic subject-to-sample response (SSTSR) profile consisting of 1,548 (18 × 86) paired chill elements. The multivariate statistical correlation of measured chill values with acoustic features and personal attributes was modeled by using random forest (RF) regression in a supervised manner, which was compared with linear partial least square (PLS) and non-linear support vector machine (SVM). The RF model exhibits possessed strong fitting ability (*r*_F_^2^ = 0.857), good generalization capability (*r*_P_^2^ = 0.712), and out-of-bag (OOB) predictability (*r*_O_^2^ = 0.731) as compared to SVM and, particularly, PLS, suggesting that the RF-based QPPR approach is able to explain and predict the emotional change upon musical arousal. It is imparted that there is an underlying relationship between the acoustic physical property of music and the physiological reaction of the audience listening to the music, in which the *rhythm* contributes significantly to emotional response relative to *timbre* and *pitch*. In addition, individual differences, characterized by personal attributes, is also responsible for the response, in which *gender* and *age* are most important.

## 1 Introduction

Emotion shapes how we think, feel, and behave (Etkin et al., 2015), which represents conscious mental reactions (such as anger or fear) subjectively experienced as strong feelings usually directed toward a specific object and typically accompanied by physiological, psychological, and behavioral changes in the body (Oatley and Jenkins, 1992; Cabanac, 2002). Over the past decades, answers to the question “what are human emotions for?” have stimulated highly productive research programs on diverse mental phenomena in psychology and behavioral science, which also plays an important role in social functionality (Niedenthal and Brauer, 2012). Currently, emotion has attracted a lot of interests in the neuroscience, biology, and medicine communities (Blanchard and Blanchard, 1988; Barrett and Satpute, 2019).

Music has long been thought to influence human emotions, which can involve cognitive processes influencing emotion and diverse emotionally evoked processes through different ways (Pearce, 2023). Traditionally, the influence of music on emotions has been described as dichotomous, a representation of external reality, or catharsis, a purification of the soul through an emotional experience (Moore, 2017), but recent neuropsychology progression has unraveled a different picture about the molecular and physiological mechanism of emotional response to music. For example, neuroimaging revealed that music can simulate the release of dopamine from the brain to modulate reward experiences (Mas-Herrero et al., 2018); it showed a causal role of dopamine in musical pleasure and indicated that dopaminergic transmission might play different or additive roles than the ones postulated in affective processing so far, particularly in abstract cognitive activities (Ferreri et al., 2019). In addition, human emotion induced by music should be treated as feelings that integrate cognitive and physiological effects, which may be accounted for by widely different production rules (Scherer, 2004).

Human emotion is a physiological event, whereas music is a structured sequence of sounds in the objective world, which can be characterized by a variety of physical quantities such as frequency, period, intensity, and duration; their specific arrangement can result in multiple vocal effects such as pitch, timbre, and rhythm to define the acoustic features of music (Nanni et al., 2016). Previously, we proposed a hypothesis that there is a complex, implicit, and intrinsic relationship between the sound features of music and human emotion aroused by the music and successfully modeled the relationship on audiences who are familiar with the listened music (Su and Zhou, 2021, 2022). Recently, we also described a new concept termed the musical protein that maps the time sequence of music onto the spatial architecture of proteins (Su and Zhou, 2024). Here, we are curious if the relationship can be established for the audiences who have never heard the music before but there is a common cultural background between the audiences and the music. In this respect, we herein adopted the Shu culture as the cultural background to explore so. Shu is an ancient geographical concept located in Southwest China, where today covers the entire Sichuan province and most area of Chongqing province, and is partially extended to their surrounding regions such as Guizhou, Yunnan, and Tibet provinces. Shu culture has more than 4,000 years of history and is currently recognized as one of three major origins of the Chinese nation (Fei, 2017; Tan, 2021).

In this study, we further proposed a new notion termed quantitative physics–physiology relationship (QPPR) to link the physical quantity of nature and the physiological behavior of humans in a statistical learning framework, which is a terminological counterpart of the quantitative structure–activity relationship (QSAR), a statistical learning strategy that has been widely used in the biology and medicine (Perkins et al., 2003). Over the past decade, our group has addressed considerable efforts on the QSAR studies (Zhou et al., 2013, 2021; Zhou et al., 2021; Lin et al., 2023), and we herein, for the first time, proposed the term QPPR to extend the applications of QSAR strategy in the physiology and neuroscience. In this study, the QPPR was created for the emotional response of people to traditional/ancient music in the Shu area by using sophisticated machine learning approaches. The resulting QPPR models were compared and analyzed systematically, and their interpretability and predictive power were also discussed in detail. The current study can be regarded as an attempt to understand the indirect, implicit, and underlying link between the material world and human physiology.

## 2 Materials and methods

### 2.1 Overview of QPPR modeling procedure

**Figure 1:**
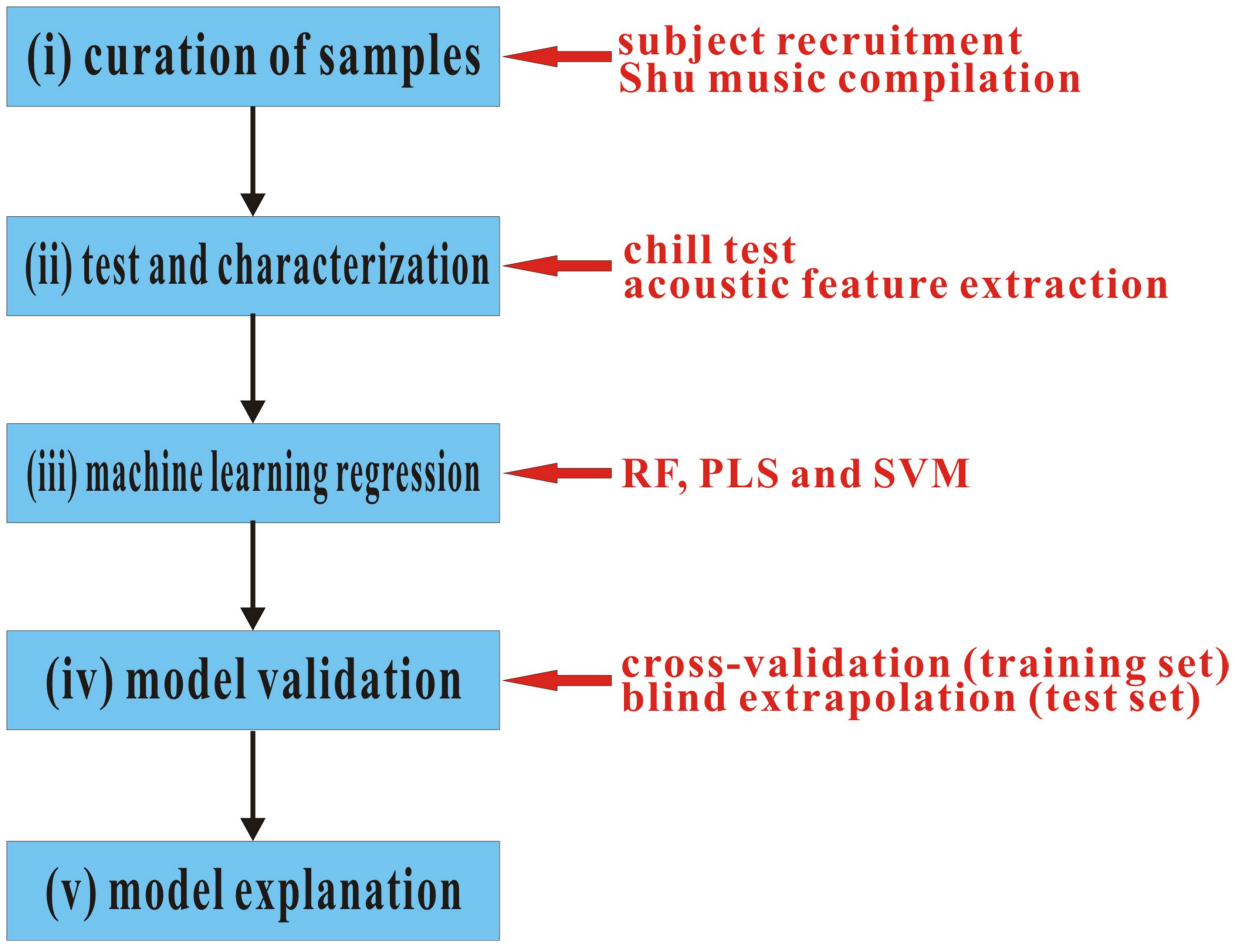
Schematic representation of the QPPR modeling procedure.

In this study, the QPPR modeling procedure was schematically shown in Figure 1, which can be roughly divided into five steps: (i) a subject group was recruited, which includes 18 persons with a wide age range, typical physical traits of the Han nationality, and normal weights as well as diverse education experiences; in addition, an in-house repertoire consisting of 86 traditional Shu music was compiled. (ii) A chill test was carried out to detect the systematic emotional response of 18 subjects to 86 music, which measured the heart rate change of a tested person when listening to a music track. A total of 15 acoustic features were extracted from the musical track. (iii) Supervised regression implemented by three machine learning methods, including RF, PLS, and SVM, was used to statistically correlate the emotional response with acoustic features in both linear and non-linear manners. (iv) Cross-validation on the training set and blind extrapolation on the test set were performed to examine the internal stability and external generalization capability of created machine learning regression models, respectively. (v) The created models were further analyzed and explained in a statistical point of view to give insights into the physical and physiological significance as well as the complex, intrinsic, and implicit relationship between them underlying the models.

### 2.2 Acoustic feature characterization

The musicological genre was used to represent the acoustic features of music, which was classified into timbre, rhythm, and pitch (Tzanetakis and Cook, 2002; Lee et al., 2006) and can be analyzed in the frequency domain (FD) with short-time Fourier transform (STFT) to derive the spectral property of soundtrack over music play (Serwach and Stasiak, 2016). The timbre represents the general perception of musical sound and characterizes the subjective aspects of music such as noisiness and brightness. The rhythm is a regularly repeated pattern of sounds or beats used in music and characterizes the sum of beat strengths over a unit session, which involves multiple salient peaks to indicate dominant and secondary beats in the music. The pitch is related to the vibration frequency of sound waves during music play and is the perceptual correlate of the rate of repetition of a periodic sound (McPherson and McDermott, 2018). Here, we adopted 15 acoustic features to characterize the physical sound property of the music track (Table 1); their details can be found in our previous research studies (Su and Zhou, 2021, 2022; Su and Zhou, 2022).

**Table 1:** Musicological genres and acoustic features used to characterize the sound property of music tracks.

Feature	Genre	Abbreviate
Spectral centroid	Timbre	SC
Spectral rolloff	Timbre	SR
Spectral flux	Timbre	SF
Zero crossing	Timbre	ZC
Mel-frequency cepstral coefficient	Timbre	MFCC
Relative amplitude of salient peaks	Rhythm	RASP
Ratio of peak amplitudes	Rhythm	RPA
Period of first and second peaks	Rhythm	PFSP
Sum of all rhythm histograms	Rhythm	SARH
Amplitude of folded maximum peak	Pitch	AFMP
Amplitude of unfolded maximum peak	Pitch	AUMP
Period of folded maximum peak	Pitch	PFMP
Period of unfolded maximum peak	Pitch	PUMP
Interval between the two highest peaks	Pitch	ITHP
Sum of all pitch histograms	Pitch	SAPH

### 2.3 Chill

Chill represents the body reaction experienced when the audience listens to music, which is generally associated with positive musical arousal (Fleuriana and Pearce, 2020). Grewe et al. (2005, 2007, 2009a) have addressed significant works on the music-elicited chill effect and found that the chill is a repeatable physiological phenomenon simulated by a specific musical event over several days for one audience, although not all persons can experience an obvious chill process when enjoying music. Previously, we employed the heart rate (*HR*) as a measurable indicator of chill, which can be quantitatively tested using a protocol described previously (Su and Zhou, 2021). Blood and Zatorre found that the *HR* was raised significantly during chill than many other chill parameters such as skin temperature and conductivity (Blood and Zatorre, 2001). In addition, the *HR* chill test is simple, non-invasive, and readily operable. However, it is worth noting that the *HR* cannot be directly equated with human emotion, which is just a measurable physiological quantity to indirectly reflect the elusive emotional response to music.

All tests were performed in a personal session to guarantee that subjects could concentrate on the music sample. The subject was set away from the acoustic source, and then, the heartbeats were recorded over the musical duration (*NH*^music^). For placebo control, the heartbeats were also counted for the same duration, but no music was played (*NH*^control^). Consequently, the chill of one subject in response to music can be expressed as a relative value of *HR* (*rHR*):


(1) $$rHR=\frac{NH^{\mathrm{music}}-NH^{\mathrm{control}}}{NH^{\mathrm{control}}}$$

where *rHR* > 0, = ~0, and < 0 indicate the positive, neutral, and negative emotional responses of the subject to music, respectively.

A total of 18 persons, including nine males and nine females, were used as subjects to perform the testing of emotional response to Shu music. All these subjects are the local people of Sichuan province, China, the core area of Shu civilization, but they have not experienced professional music education. Therefore, they share a similar cultural background with Shu music, but the testing would not be biased by their personal experience. In addition, these subjects have a wide age range between 18 and 72 years old, typical physical traits of the Han nationality, that is, regular statures (162–178 cm for men and 151–168 cm for women) and normal weights (54–76 kg for men and 42–57 kg for women), as well as diverse education experiences (from primary school or lower to master degree or higher) (Table 2). All procedures performed in this study were in accordance with the ethical standards of the institutional research committee and with the 1964 Helsinki Declaration and its later amendments or comparable ethical standards.

**Table 2:** Personal attributes of 18 subjects.

Subject	Gender*^a^*	Age (years)	Stature (cm)	Weight (kg)	Education*^b^*
P1	0	22	177	68	2
P2	0	49	166	65	1
P3	0	65	162	54	1
P4	0	19	171	59	2
P5	0	31	169	61	3
P6	0	38	168	72	1
P7	0	18	174	74	1
P8	0	27	178	76	2
P9	0	54	165	59	0
P10	1	20	167	48	1
p11	1	62	153	42	0
p12	1	43	158	53	1
p13	1	52	157	48	1
p14	1	29	163	51	3
P15	1	58	157	50	0
P16	1	72	151	46	0
P17	1	36	161	54	1
P18	1	23	168	57	2

^a0, male; 1, female. b0, primary school or lower; 1, junior high or higher school; 2, bachelor or associate degree; 3, master degree or higher.^

### 2.4 QPPR machine learning regression

Random forest (RF) regression (Breiman, 2001) was applied to create the QPPR models between the emotional response of subjects to music samples and the acoustic features of these samples. In addition, other two sophisticated machine learning methods were also performed for comparison purposes, including a linear partial least squares (PLS) regression (Geladi and Kowalski, 1986) and a non-linear support vector machine (SVM) regression (Cortes and Vapnik, 1995). In this study, all the regression modeling was implemented by an in-house Matlab package ZP-explore (Zhou et al., 2009), which has previously been widely used in the QSAR studies of biological and medicinal issues (Liu et al., 2022; Zhou et al., 2022).

RF is an ensemble of *n* unpruned trees [*t*1(***x***), *t*2(***x***), …, t*n* (***x***)], where ***x*** is an *m*-dimensional vector of inputs [*x*1, *x*2, …, *xm*] (Khan et al., 2020). Each tree is trained by a bootstrap sampling on training data and then conducts prediction by averaging the outputs. The method only considers a random subset of inputs, instead of all the inputs at each node of each tree. The strength of these trees in RF was maximized, whereas the correlation between them was minimized. The size of the variable subset is a fixed number. RF has many advantages to improve its practicability and feasibility for machine learning regression (Bylander, 2002), such as out-of-bag (OOB) validation and variable importance.

PLS is a widely used regression method to model the linear relationship between the multivariate independent inputs (acoustic features) and a single dependent output (emotional response) (Abdi, 2010). It can overcome the collinearity issue and is especially suitable for treating small sample sizes and large input number. Here, a latent variable should be excluded from the PLS regression if the increase in cumulative cross-validation coefficient of determination *r*_C_^2^ was below 0.097 by introducing the variable, as it could not explain any significant physics–physiology trend (Tian et al., 2009).

SVM is based on statistical learning theory (SLT) and applied to structural risk minimization, instead of traditional empirical risk minimization, which is applicable to solving the problems of small samples, high dimension, and strong collinearity (Noble, 2006). The method transforms quadratic convex programming into a dual problem via the Lagrange approach and then utilizes the kernel function to perform the inner product operation in a high-dimensional Hilbert space. Here, the radial basis function (RBF) was used as the kernel of SVM (Ding et al., 2021).

### 2.5 QPPR model validation

The whole data panel was split into an internal training set and an external test set (Gramatica, 2007). The former was used to internally build QPPR regression models in a fitting manner, whereas the latter was employed to externally validate the built models in a blind extrapolation manner (Andersson et al., 1998). The underlying goal of splitting was to ensure that both the training and test sets separately span over the whole space occupied by the entire sample dataset and that the samples in the two sets are not too dissimilar (Tropsha et al., 2003).

#### 2.5.1 Internal validation

The model stability was validated by 10-fold cross-validation based on the supervised training set.

#### 2.5.2 External validation

The model predictability was validated by blindly extrapolating to the test set.

The regression performance can be measured quantitatively by using the coefficient of determination (COD) deriving from fitting on the training set (*r*_F_^2^), cross-validation on the training set (*r*_C_^2^), and prediction on the test set (*r*_P_^2^), as well as corresponding root-mean-square errors (RMSE_F_, RMSE_C,_ and RMSE_P_, respectively). In addition, RF has also an additional OOB validation to derive *r*_O_^2^ and RMSE_O_. Their definitions can be found in our previous publications (Zhou et al., 2013; Khan et al., 2020).

### 2.6 Compilation of traditional Shu music repertoire

Our group is located in Chengdu City, the capital of Sichuan Province, which is the core region of the Shu area. Over the past decade, we have focused on the Shu civilization for a long time and addressed significant concerns about the arts and music of Shu culture. The Shu area has a variety of traditional music forms with typical local style, which are mainly folk songs, divine songs, antiphonal songs, paddy songs, rolling board, minore, and chant as well as those of minority nationalities such as Tibetan, Qiang, and Yi in the western Sichuan. Ancient Shu culture is one of the oldest art systems in China, which includes various music genres that have been inherited for generations. The genres are influenced by many cultural factors over the Shu history (>4,000 years). However, most ancient music has been lost during the long history and only few are still recorded today. Considering that familiarity with the music has a significant impact on emotional response (Grewe et al., 2009b), we herein only selected those traditional/ancient Shu music that are familiar to most Shu people. In addition, the selected music should be diverse in terms of their genre and style. In this way, a total of 86 traditional/ancient Shu music were compiled to define a distinct repertoire, in which most were published today by 50–200 years, but there are also a number of music that were composed thousands of years ago to Pre-Qin (earlier than B.C. 221). Moreover, these music samples were collected from different subareas of Shu and also cover a wide range including lyric, affectionate, cheerfulness, sonorous, and so on. Here, an in-house repertoire of the 86 traditional/ancient Shu music samples is listed in Supplementary Table S1.

## 3 Results and discussion

### 3.1 Systematic profiling of the human emotional response to Shu music

**Figure 2:**
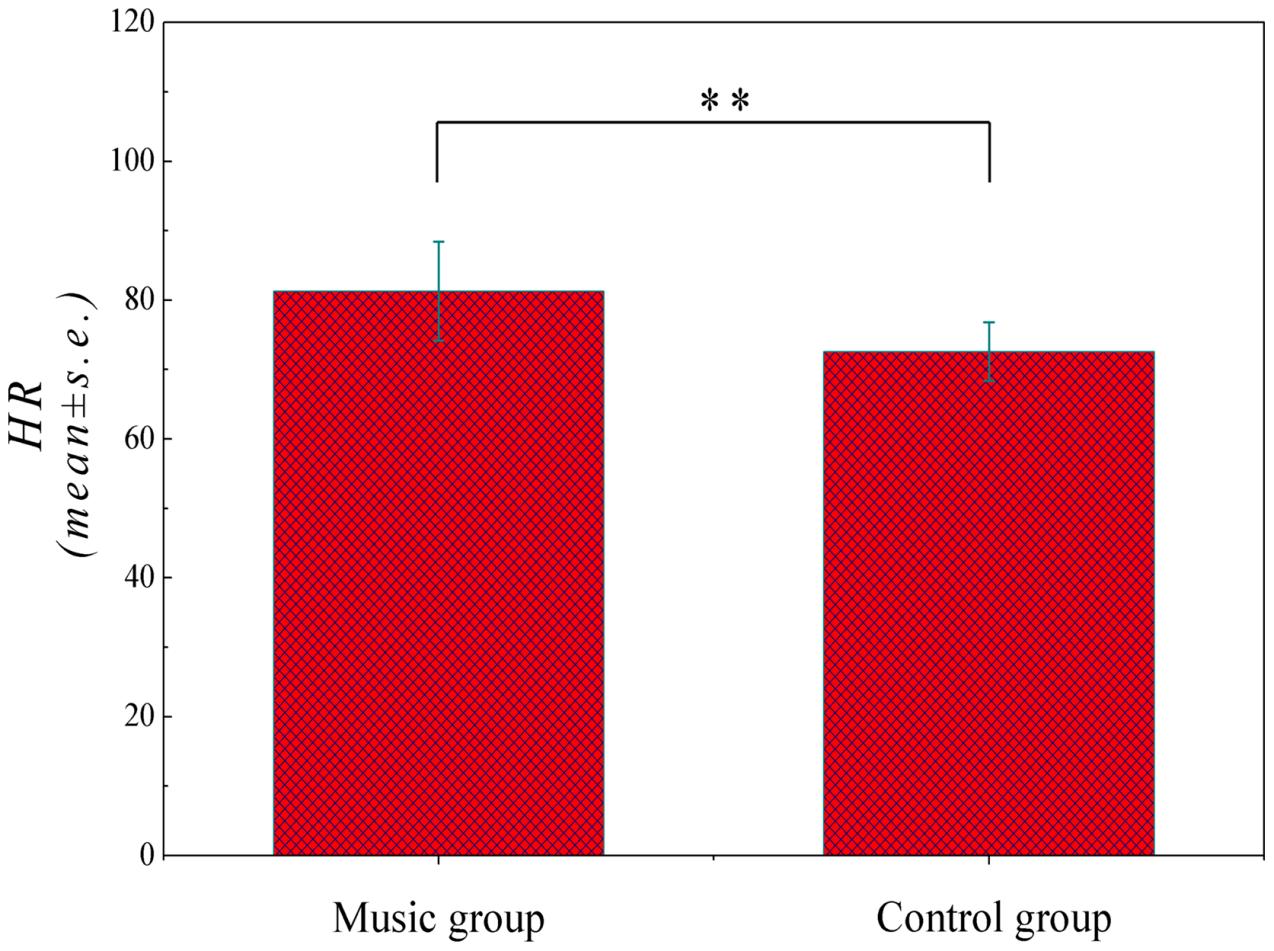
Average *HR* values (*mean* ± *s.e.*) between control and music (*p* < 0.05).

The systematic chill profile of 18 audience subjects in response to 86 music samples was created as characterized by the *HR* values derived from Equation 1, consequently obtaining 1,548 subject–sample pairs. As can be seen in Figure 2, the average *HR* values (*mean* ± *s.e.*) of control and music groups are compared in a histogram form to unravel a significant difference between them (*p*-value <0.05), thus imparting that the human emotion can be influence by musical arousal with average HR increase from 72.6 ± 4.2 to 81.3 ± 7.1 per min. Specifically, the error bar also increases from 4.2 to 7.1, indicating that the music exhibits differential effects on distinct persons, albeit the overall trend is increased consistently.

**Figure 3:**
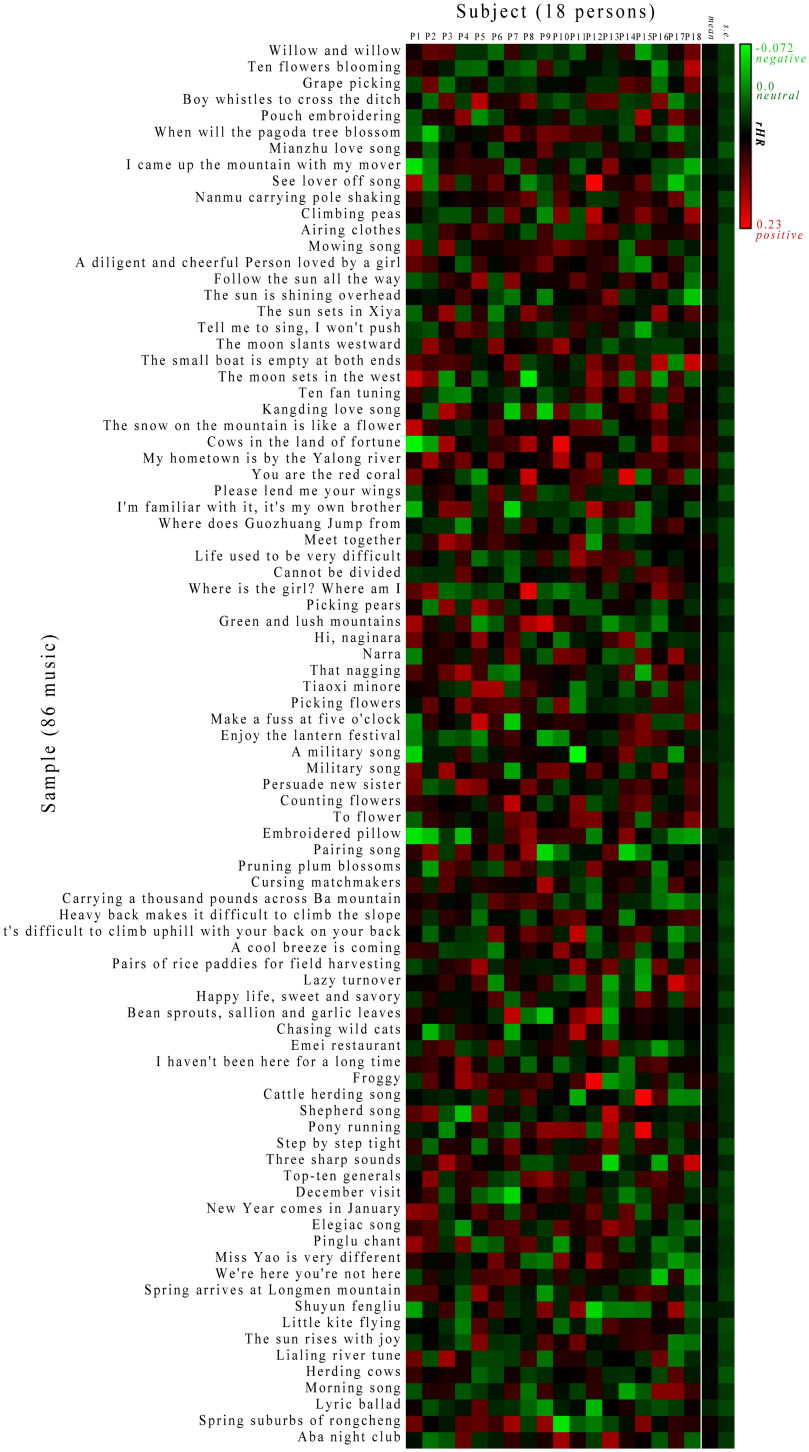
Systematic subject-to-sample response (SSTSR) profile characterized by *rHR*. The red, black, and green indicate positive, neutral, and negative responses (*rHR* > 0, = 0 and < 0), respectively.

Considering that the individual bias may have an effect on the absolute value of *HR* test results, the relative *rHR* value was adopted here as an unbiased measure to compare different tests by eliminating the individual bias. On this basis, a systematic subject-to-sample response (SSTSR) profile was created to represent varying responses in different colors as visualized in Figure 3 (Supplementary Table S2). Evidently, the chill is common as most responses are highly or moderately positive for subjects during the music listing session. This is because all these subjects are local Shu people who share a similar cultural background, which is responsible for chill (Grewe et al., 2009b). In addition, some neutral effects can be observed in the profile, as the emotional activity represents human mental behavior that is very subjective; only ~65% of subjects have been reported to experience chill during a session with either their own favorite music or selected music (Ferreri et al., 2019). In addition, few negative responses in the profile represent a peaceful period during which the chill is reduced relative to placebo.

### 3.2 Comparative analysis of human physiological response to Shu music

**Figure 4:**
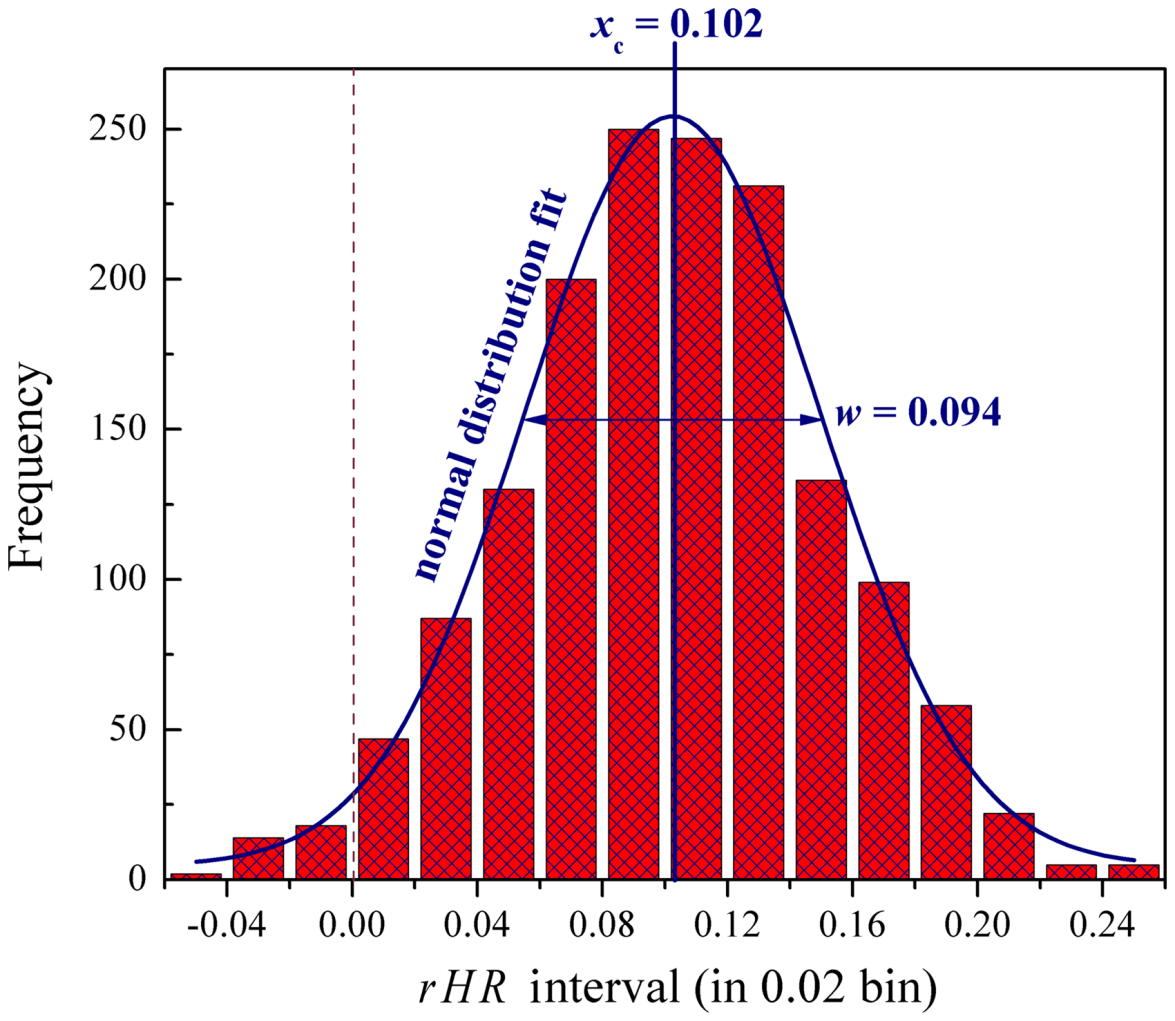
Histogram distribution of 1,548 pairwise observed *rHR* values ranging in the interval [−0.06, 0.26] and 0.02 bin. The distribution can be well fitted by using a Gauss normal function with peak at *x*_c_ = 0.102 and radial width *w* = 0.094.

The 1,548 pairwise observed *rHR* values are further plotted as a histogram in terms of their distribution ranging in the interval [−0.06, 0.26] and 0.02 bin. As shown in Figure 4, most observed values are located in *rHR* > 0, whereas there are only very few with *rHR* < 0, indicating that the subjects generally have a moderate or significant response to these samples, namely physiology of subjects can be affected by music listening, thus stimulating an emotional resonance between them. This is expected if considering that the Shu music is mostly cheerful and inspiring, which could promote audience enthusiasm. For example, a previous study suggested that traditional Shu music can be used as an effective assistant tool in music therapy to treat patients with moderate to mild depression (He and Zhu, 2020). In addition, the *rHR* histogram distribution can be well fitted by using a Gauss normal function with a peak at *x*_c_ = 0.102 and radial width *w* = 0.094, which implies that such physiological response, from a statistical point of view, is not fully random, which would involve certain regularities.

**Figure 5:**
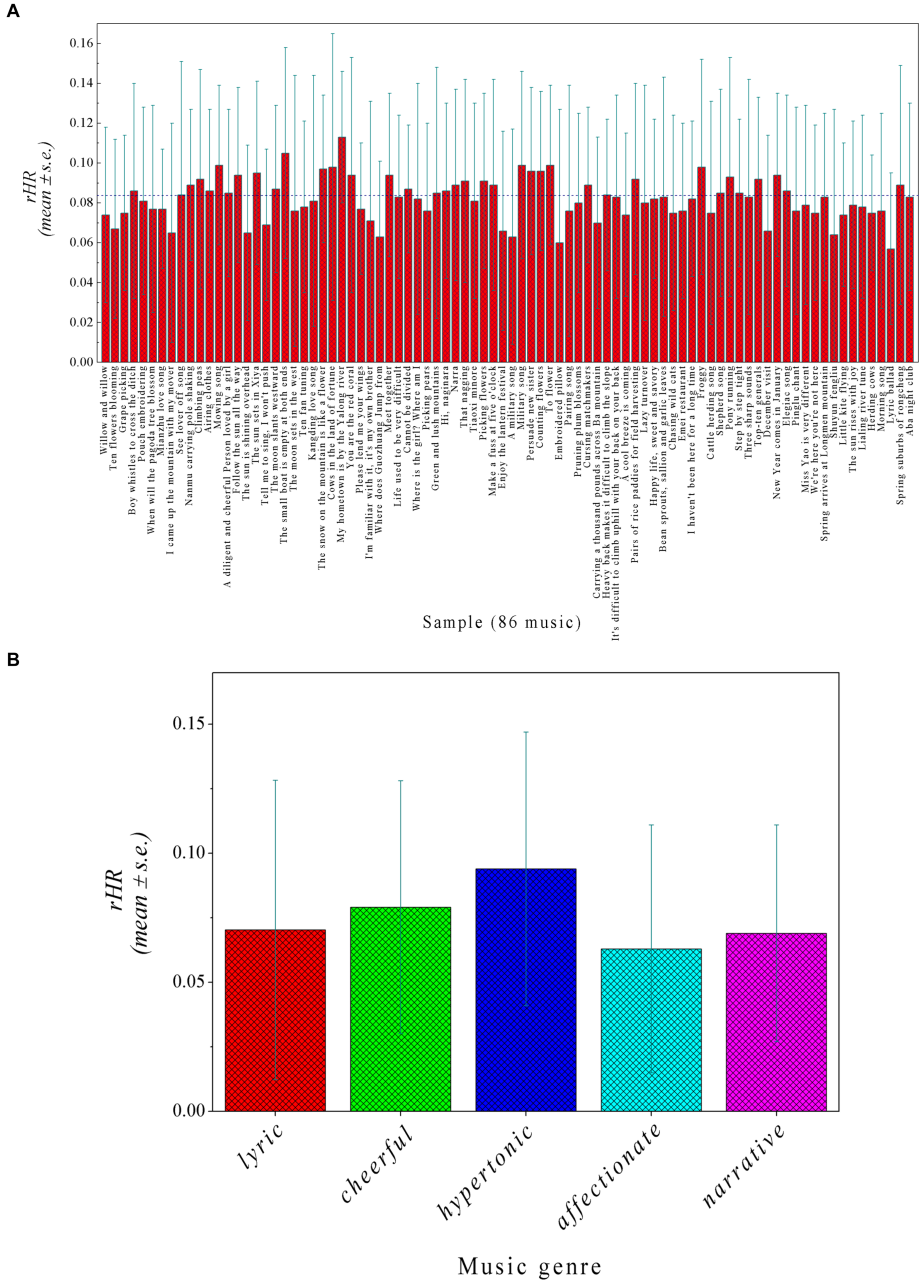
**(A)** *rHR* values (*mean* ± *s.e.*) of each music sample over 18 subjects. **(B)** *rHR* values (*mean* ± *s.e.*) between five music genres (*lyric*, *affectionate*, *cheerful*, *hypertonic,* and *narrative*).

The *rHR* values change from 0.057 to 0.113 over the 86 music samples (Figure 5A), indicating that different genres and styles of music may have distinct effects on the physiological behavior of audiences. The *rHR* > 0 also indicates that Shu music can generally address a consistent effect on subjects. This is quite different from, for example, the Western music that has been found to address diverse physiological features in audiences (Su and Zhou, 2022). In particular, these music samples were observed to modulate chill for most subjects. For instance, *rHR* = ~0.05 imparts a neutral influence of *Lyric Ballad* and *Embroidered Pillow* on these subjects; the two music represent a narrative genre that cannot change one mind significantly and thus have no substantial effect on subjects. Some other cheerful and hypertonic samples like *My Hometown is by the Yalong River* and *The Moon Sets in the West* can arouse subjects with *rHR* = >0.1. Figure 5B further compares the *rHR* values between five genres (*lyric*, *affectionate*, *cheerful*, *hypertonic,* and *narrative*), unraveling that the musical genre is also responsible for chill. For example, the *cheerful* and *hypertonic* can contribute positively to emotion, while the *lyric*, *affectionate,* and *narrative* have only a neutral or modest effect on the chill. Moreover, different music styles (minore, folk song, divine song, love song, paddy song, and Jew’s harp) also exhibit differentiated influences on chill, although its effect on chill is weaker than music genre. The influence of other factors (such as subarea and author) on human physiological behavior was not discussed here due to the lack of sufficient samples to derive statistically significant conclusions.

### 3.3 Development, optimization, and validation of RF-based QPPR models

The 86 music samples were randomly divided into an internal training set of 60 samples for regressively fitting the QPPS model, and an external test set of the remaining 26 samples for blindly validating the model (Gramatica, 2007). Random forest (RF) was used to relate the physical acoustic quantity (acoustic feature) of Shu music with the physiological emotional response (chill) of subjects. In the first modeling, we only focused on the chill change upon music variation but did not consider the individual bias between the 18 subjects. Therefore, the dependent variable (*y*_i_) was assigned for each sample *i* with the average *rHR* value over 18 subjects, while the independent variables are the 15 acoustic features (*x_i_*^1^, *x_i_*^2^, *x_i_*^3^ … *x_i_*^15^) associated with the sample *i*. On this basis, a QPPS model was created by RF regression to statistically correlate between (*x_i_*^1^, *x_i_*^2^, *x_i_*^3^ … *x_i_*^15^) and (*y*_i_) over the 60 training samples in a supervised manner. During the RF modeling process, a grid search was carried out to optimize the best parameter combination [*mtry*/all, *ntree*]. The *ntree* is the number of unpruned decision trees in RF, with a search range between 10 and 100, while the *mtry*/all is the ratio of the random variable subset to the complete variable set in each tree, ranging between 0.1 and 1. As can be seen, the RF modeling is very unstable when both the *mtry*/all and *ntree* are small, characterized by a large RMSE_C_ error by internal cross-validation. However, the RMSE_C_ decreases fast upon the increase of *mtry*/all and *ntree*; the modeling would become roughly stable if *mtry*/all >0.3 and *ntree* > 20, and the RMSE_C_ reaches a minimum 0.032 at *mtry*/all = 0.8 and *ntree* = 75, which were then utilized to build the RF-based QPPS model (I). It is suggested that high performance of modeling on a training set is a necessary but not a sufficient condition for a supervised model to have high predictive power; they also emphasized that external validation is the only way to establish a predictive model (Golbraikh and Tropsha, 2002a). In this respect, the model (I) was validated by blindly extrapolating to the test set. Here, the resulting model statistics are tabulated in Table 3. It is evident that the QPPS model has a strong fitting ability on the training set (*r*_F_^2^ = 0.740) and good internal stability by cross-validation (*r*_C_^2^ = 0.617). In addition, the model also possesses a moderate external predictability on the test set (*r*_P_^2^ = 0.529), which is considerably higher than the randomly expected value (*r*_P_^2^ = 0), but does not achieve a powerful predicted result (*r*_P_^2^ > 0.6) as suggested by (Golbraikh and Tropsha, 2002b). In addition, the RF OOB validation also gave a similar predictive profile with the blind validation on test set (*r*_O_^2^ = 0.537 versus *r*_P_^2^ = 0.529), confirming that the model (I) has only a moderate generalization ability on extrapolation.

**Table 3:** Statistics of different QPPS models built by RF, PLS, and SVM.

Method	Model	Parameter*^a^*	Training set							Test set		
			Size*^b^*	*r*_F_^2^	*r*_C_^2^	*r*_O_^2^	RMSE_F_	RMSE_C_	RMSE_O_	Size*^b^*	*r*_P_^2^	RMSE_P_
RF	(I)	*ntree* = 75 *mtry*/all = 0.8	60	0.740	0.617	0.537	0.024	0.032	0.037	26	0.529	0.039
RF	(II)	*ntree* = 80 *mtry*/all = 0.7	1,100	0.857	0.796	0.731	0.072	0.081	0.090	448	0.712	0.095
PLS	(III)	*NL* = 5	1,100	0.613	0.478	/^c^	0.112	0.138	/	448	0.316	0.157
SVM	(IV)	*ε* = 0.15\u00B0*C* = 100 *σ*^2^ = 27	1,100	0.826	0.737	/	0.079	0.095	/	448	0.620	0.112

^aParameters used in machine learning regression. bNumber of training samples used to develop the model or test samples used to validate the model.^

**Figure 6:**
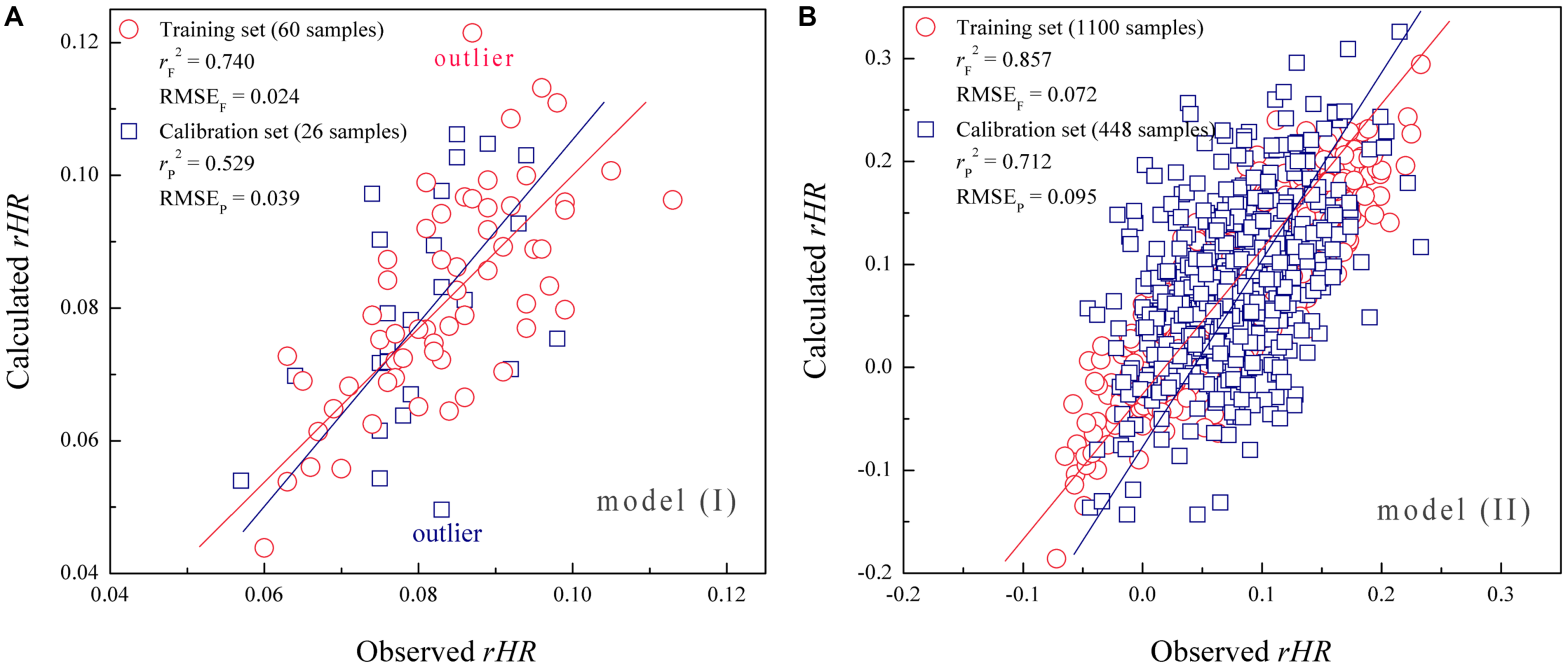
**(A)** Scatter plot of model **(I)**-calculated versus measured average *rHR* values over 86 data points (including 60 training samples and 26 test samples). **(B)** Scatter plot of model **(II)**-calculated versus measured *rHR* values over 1,548 data points (including 1,100 training samples and 448 test samples).

The scatter plot of model (I)-fitted versus measured average *rHR* values over 60 training samples and model (I)-predicted versus measured average *rHR* values over 26 test samples is shown in Figure 6A. Evidently, these data points are roughly distributed around the slope fitted through them, but the distribution is not very even, exhibiting a considerable variation. In particular, there are few outliers that can be observed in the plot, which deviate significantly from the slope, indicating that some implicit information underlying the emotion–response relationship cannot be captured and unraveled by the model (I). We think this is because the model did not consider the individual bias; that is, the physiological effect can be quite different varying over subjects when listening to the same music sample. Previous studies also observed that individual difference has a statistically significant variation across the mental behavior of different audiences (Su and Zhou, 2021). Therefore, we further adopted all the 1,548 *rHR* values to rebuild the RF-based QPPR model (II), which represent the full profile of the emotional response of 18 subjects to 86 samples (18 × 86 = 1,548) shown in Figure 3, in which each *rHR* value characterizes the chill of a specific subject when listening to a given music, thus the value involves both music and subject information. Here, we randomly selected 1,100 data from the 1,548 *rHR* values as a training set to build the model (II), while other 448 data were engaged in a test set to blindly validate the model. A total of 20 independent variables were considered, including 15 acoustic features (*x_i_*^1^, *x_i_*^2^, *x_i_*^3^ … *x_i_*^15^) associated with the given sample *i* as well as 5 personal attributes (*x_j_*^16^, *x_j_*^17^, *x_j_*^18^ … *x_j_*^20^) associated with the specific subject *j*, which were then correlated with the dependent variable (*y*_i_) of chill *rHR* values by using RF modeling, consequently resulting in the model (II), of which the obtained statistics are tabulated in Table 3. As might be expected, the model (II) was improved substantially relative to the model (I) in fitting ability, internal stability, and external predictability, with *r*_F_^2^ = 0.857, *r*_C_^2^ = 0.796, *r*_P_^2^ = 0.712, and *r*_O_^2^ = 0.731 for model (II), which are considerably or moderately better than that *r*_F_^2^ = 0.740, *r*_C_^2^ = 0.617, *r*_P_^2^ = 0.529, and *r*_O_^2^ = 0.537 for model (I), which will meet the ‘gold standard’ suggested by Golbraikh and Tropsha (2002b) for a reliable and predictive regression model, that is, *r*_F_^2^ > 0.8, *r*_C_^2^ > 0.6, and *r*_P_^2^ > 0.6. Moreover, it is seen in Figure 6B that all the data points are basically distributed evenly around the slope in the scatter plot of calculated versus measured *rHR* values, and non-outlier can be observed in the scatter, confirming that the model (II) has a good performance as compared to model (I), which can be used to well explain the implicit music–response relationship by considering the individual difference in a supervised manner and then address predictive extrapolation on those untrained data points.

### 3.4 Analysis and explanation of RF-based QPPR model (II)

**Figure 7:**
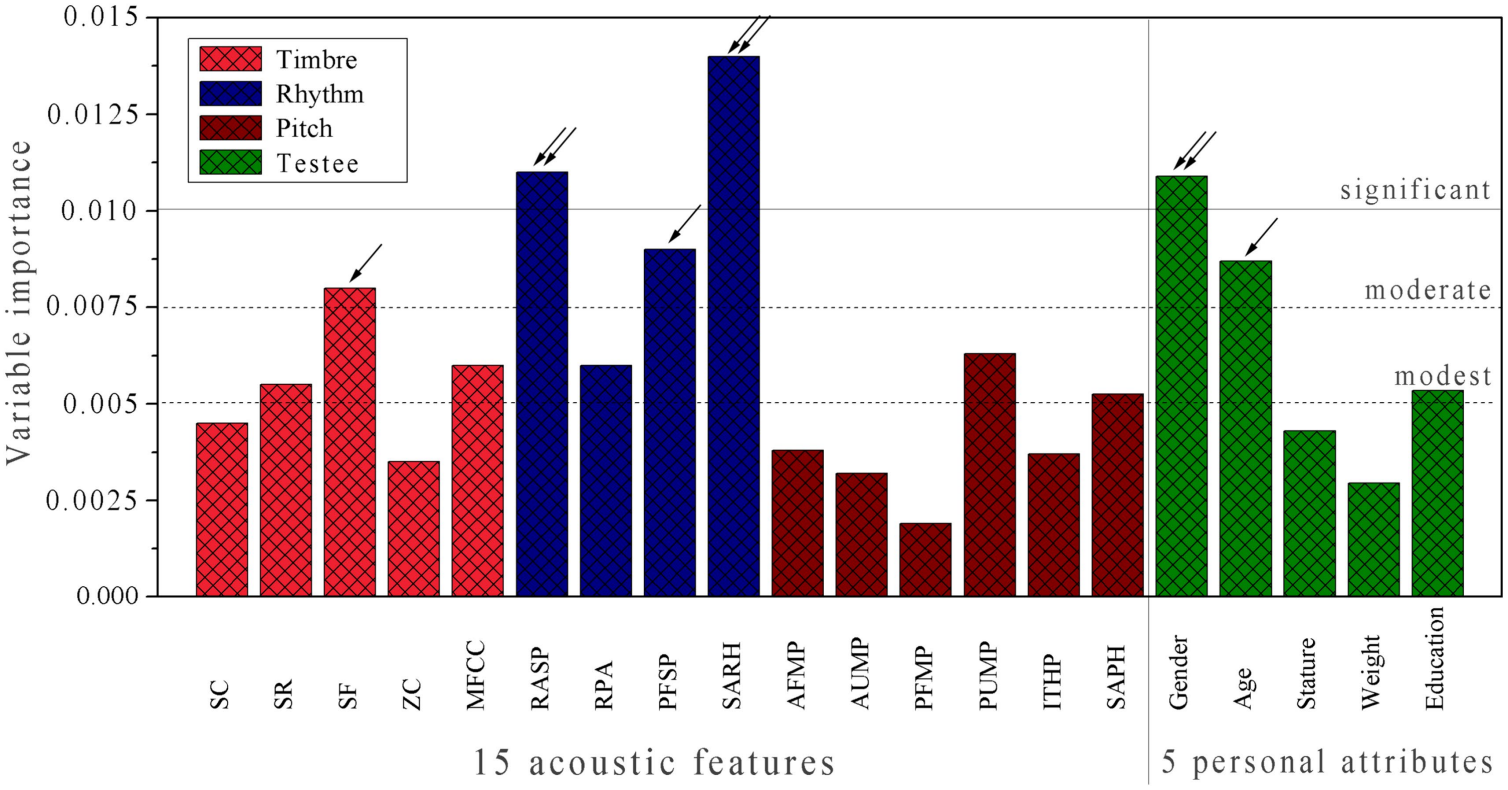
Variable importance (VI) profile of RF-based model (II), which characterizes the independent contribution of each of 15 acoustic features and 5 personal attributes to the model.

The RF-based model (II) exhibits a good performance in fitting ability, internal stability, and external predictability as compared to model (I), imparting that, in addition to the acoustic property of music samples, the individual difference has also a substantial influence on chill. This is not unexpected because the emotional response is a physiological behavior that can be differentiated significantly by varying over different subjects. Therefore, we herein further examined the variable importance (VI) in the model (II). Each (*i*) of the 15 acoustic features and 5 personal attributes was removed from the model and then rebuilt a new model (∆*i*) with the remaining 19 variables. Consequently, the generalization ability degradation upon the variable removal can be expressed as: VI = RMSE_P_^(*i*)^ – RMSE_P_, where the RMSE_P_ and RMSE_P_^(*i*)^ are the root-mean-squares error of prediction on the test set by using model (II) and model (∆*i*), respectively. This is a general method to systematically determine the statistically independent contribution of each variable to a model, which was used to characterize variable importance (VI). It is revealed from Figure 7 that: (a) The contribution of three acoustic classes to the model increases in the order: *pitch* < *timbre* < *rhythm* (VI*^Pitch^* < VI*^Timbre^* < VI*^Rhythm^*), in which the difference between VI*^Pitch^* and VI*^Timbre^* is not very significant, and the contribution primarily arises from *rhythm*. (b) The importance varies significantly over the 5 personal attributes, in which *gender* and *age* are mostly associated with emotional response (VI*^Gender^* > 0.010 and VI*^Age^* > 0.0075), and *education* contributes moderately to the response (VI*^Education^* > 0.005), but other two personal physical attributes *stature* and *weight* have only a modest effect on the response (VI*^Stature^* and VI*^Weight^* < 0.005). (c) In general, the four acoustic features SF, RASP, PFSP, and SARH as well as two personal attributes *gender* and *age* are primarily responsible for emotional response, with VI > 0.0075, whereas most *pitch* features and certain *timbre* features can only contribute limitedly to the model, which can be regarded as secondary factors influencing the response.

### 3.5 Comparison of RF with linear PLS and non-linear SVM in QPPRs

We further rebuilt the QPPR model by using linear PLS and non-linear SVM based on the 1,548 *rHR* values and with 15 acoustic features plus 5 personal attributes, consequently resulting in models (III) and (IV), respectively. Their model parameters, including the number of latent variables (*NL*) for PLS and kernel parameters *ε*, C, and *σ*^2^ for SVM were optimized systematically through grid search; the obtained parameters and resulting statistics for the two models (III) and (IV) are tabulated in Table 3. By comparison, the fitting ability (*r*_F_^2^ = 0.613), internal stability (*r*_C_^2^ = 0.478) and, particularly, external predictability (*r*_P_^2^ = 0.316) of PLS-based model (III) are considerably lower than that of RF-based model (II), indicating that there is a significant non-linear correlation involved in the music-response relationship so that the linear PLS is unable to well treat the non-linear issue for the relationship. In addition, the model (IV) built by the widely used non-linear SVM also performed much better than PLS-based model (III) (*r*_F_^2^ = 0.826 versus 0.613, *r*_C_^2^ = 0.737 versus 0.478 and *r*_P_^2^ = 0.620 versus 0.316) and exhibited a roughly comparable profile with RF-based model (II) (*r*_F_^2^ = 0.826 versus 0.857, *r*_C_^2^ = 0.737 versus 0.796, and *r*_P_^2^ = 0.620 versus 0.712). Overall, we ordered these machine learning methods in the QPPR modeling of the music–response relationship as follows: PLS < < SVM < RF.

## Data Availability

The original contributions presented in the study are included in the article/Supplementary materials, further inquiries can be directed to the corresponding author/s.
