# Supplementary material for: Quantitative physics–physiology relationship modeling of human emotional response to Shu music
Source: Front Psychol. 2024 Oct 8;15:1351058. doi: 10.3389/fpsyg.2024.1351058 (PMC11493695; doi:10.3389/fpsyg.2024.1351058)
Supplement: Supplementary file 1 [file Data_Sheet_1.PDF]

# Supplementary Materials

**Table S1.** An in-house repertoire of 86 traditional/ancient Shu music

| Number | Music sample | Subarea in Shu area | Style | Genre | Author | Time (min) |
|---|---|---|---|---|---|---|
| 1 | Willow and willow | Weiyuan county | folk song | lyric | unknown | 3:05 |
| 2 | Ten flowers blooming | Weiyuan county | folk song | affectionate | unknown | 2:10 |
| 3 | Grape picking | Yibin city | divine song | cheerful | unknown | 2:49 |
| 4 | Boy whistles to cross the ditch | Yibin city | divine song | cheerful | unknown | 2:48 |
| 5 | Pouch embroidering | Yibin city | divine song | lyric | unknown | 2:53 |
| 6 | When will the pagoda tree blossom | Yibin city | divine song | sonorous | Zurong Yu | 2:10 |
| 7 | Mianzhu love song | Mianzhu city | folk song | affectionate | unknown | 3:28 |
| 8 | I came up the mountain with my mover | Mianzhu city | folk song | affectionate | unknown | 3:15 |
| 9 | See lover off song | Mianzhu city | minore | affectionate | unknown | 3:18 |
| 10 | Nanmu carrying pole shaking | Luzhou city | minore | affectionate | unknown | 3:29 |
| 11 | Climbing peas | Fushun county | minore | affectionate | unknown | 3:03 |
| 12 | Airing clothes | Langzhong county | folk song | lyric | unknown | 3:19 |
| 13 | Mowing song | Pujiang county | folk song | cheerful | unknown | 3:15 |
| 14 | A diligent and cheerful Person loved by a girl | An county | folk song | lyric | unknown | 3:18 |
| 15 | Follow the sun all the way | Baqu county | folk song | lyric | unknown | 3:00 |
| 16 | The sun is shining overhead | Daba mountain zone | minore | lyric | unknown | 3:03 |
| 17 | The sun sets in Xiya | Wanyuan county | folk song | lyric | unknown | 2:50 |
| 18 | Tell me to sing, I won't push | Tomgjiang county | folk song | lyric | unknown | 2:58 |
| 19 | The moon slants westward | Dazhu county | folk song | lyric | unknown | 2:26 |
| 20 | The small boat is empty at both ends | Wanyuan county | folk song | lyric | unknown | 2:19 |
| 21 | The moon sets in the west | North Sichuan | folk song | lyric | unknown | 2:48 |

| | | | | | | |
|---|---|---|---|---|---|---|
| 22 | Ten fan tuning | North Sichuan | folk song | lyric | unknown | 2:40 |
| 23 | Kangding love song | Kangding county | love song | lyric | Wu Wenji/Jiang Dingxian | 2:53 |
| 24 | The snow on the mountain is like a flower | Mao county | Tibetan folk song | lyric | unknown | 3:48 |
| 25 | Cows in the land of fortune | Shiqu county | Tibetan folk song | lyric | unknown | 3:42 |
| 26 | My hometown is by the Yalong river | Shiqu county | Tibetan folk song | cheerful | unknown | 3:13 |
| 27 | You are the red coral | Seda county | Tibetan folk song | lyric | unknown | 3:17 |
| 28 | Please lend me your wings | Seda county | Tibetan folk song | lyric | unknown | 3:02 |
| 29 | I'm familiar with it, it's my own brother | Danba county | Tibetan folk song | lyric | unknown | 3:36 |
| 30 | Where does Guozhuang Jump from | Danba county | Tibetan folk song | lyric | unknown | 3:34 |
| 31 | Meet together | Jiulong county | Tibetan folk song | lyric | unknown | 3:07 |
| 32 | Life used to be very difficult | Liangshan city | Yi jew's harp | lyric | unknown | 3:06 |
| 33 | Cannot be divided | Liangshan city | Yi jew's harp | lyric | unknown | 2:55 |
| 34 | Where is the girl? Where am I | Liangshan city | Yi jew's harp | lyric | unknown | 2:16 |
| 35 | Picking pears | Jinyang county | Yi folk song | lyric | unknown | 2:35 |
| 36 | Green and lush mountains | Puge county | Yi folk song | lyric | unknown | 2:19 |
| 37 | Hi, naginara | Mao county | Yi folk song | lyric | Dong Fangquan | 2:13 |
| 38 | Narra | YWenchuan county | Qiang folk song | lyric | unknown | 2:05 |
| 39 | That nagging | Wenchuan county | Qiang folk song | lyric | unknown | 2:03 |
| 40 | Tiaoxi minore | Wenchuan county | Qiang folk song | lyric | unknown | 2:01 |
| 41 | Picking flowers | Nanping county | minore | cheerful | unknown | 3:12 |
| 42 | Make a fuss at five o'clock | Nanping county | minore | lyric | unknown | 3:05 |
| 43 | Enjoy the lantern festival | Nanping county | minore | lyric | unknown | 3:07 |
| 44 | A military song | Nanping county | minore | hypertonic | unknown | 3:01 |
| 45 | Military song | Nanping county | minore | hypertonic | unknown | 2:58 |
| 46 | Persuade new sister | Guangan city | antiphonal song | narrative | unknown | 2:45 |
| 47 | Counting flowers | Guangan city | antiphonal song | narrative | unknown | 2:32 |

| | | | | | | |
|---|---|---|---|---|---|---|
| 48 | To flower | Guangan city | antiphonal song | narrative | unknown | 2:20 |
| 49 | Embroidered pillow | Guangan city | antiphonal song | narrative | unknown | 2:28 |
| 50 | Pairing song | Guangan city | antiphonal song | narrative | unknown | 2:17 |
| 51 | Pruning plum blossoms | Guangan city | antiphonal song | narrative | unknown | 2:08 |
| 52 | Cursing matchmakers | Guangan city | antiphonal song | narrative | unknown | 2:10 |
| 53 | Carrying a thousand pounds across Ba mountain | Daba mountain zone | paddy song | hypertonic | unknown | 2:25 |
| 54 | Heavy back makes it difficult to climb the slope | Daba mountain zone | paddy song | hypertonic | unknown | 2:34 |
| 55 | It's difficult to climb uphill with your back on your back | Daba mountain zone | paddy song | hypertonic | unknown | 2:39 |
| 56 | A cool breeze is coming | Eastern Sichuan | paddy song | hypertonic | unknown | 2:48 |
| 57 | Pairs of rice paddies for field harvesting | Eastern Sichuan | paddy song | hypertonic | unknown | 2:55 |
| 58 | Lazy turnover | Pingshan county | paddy song | lyric | unknown | 2:00 |
| 59 | Happy life, sweet and savory | Eastern Sichuan | Tu paddy song | lyric | unknown | 3:00 |
| 60 | Bean sprouts, sallion and garlic leaves | Baqu county | paddy song | hypertonic | unknown | 2:03 |
| 61 | Chasing wild cats | Naxi zone | rolling board | hypertonic | unknown | 1:58 |
| 62 | Emei restaurant | Emeishan county | folk song | hypertonic | Liang Shangquan/Shi Xin | 2:52 |
| 63 | I haven't been here for a long time | The whole Sichuan province | folk song | cheerful | Wang Jifu/Mao Di | 3:40 |
| 64 | Froggy | The whole Sichuan province | children rhyme | cheerful | unknown | 1:40 |
| 65 | Cattle herding song | The whole Sichuan province | children rhyme | cheerful | unknown | 2:08 |
| 66 | Shepherd song | The whole Sichuan province | children rhyme | cheerful | unknown | 2:00 |
| 67 | Pony running | The whole Sichuan province | children rhyme | cheerful | unknown | 2:06 |
| 68 | Step by step tight | Anyue county | chant | hypertonic | unknown | 2:30 |
| 69 | Three sharp sounds | Anyue county | chant | hypertonic | unknown | 2:34 |
| 70 | Top-ten generals | Zigong city | chant | hypertonic | unknown | 2:25 |
| 71 | December visit | Zigong city | chant | hypertonic | unknown | 3:01 |
| 72 | New Year comes in January | Zigong city | chant | hypertonic | unknown | 3:08 |
| 73 | Elegiac song | Zigong city | chant | hypertonic | unknown | 3:03 |

| | | | | | | |
|---|---|---|---|---|---|---|
| 74 | Pinglu chant | Chengdu city | chant | hypertonic | unknown | 2:15 |
| 75 | Miss Yao is very different | Linshui county | chant | hypertonic | unknown | 2:04 |
| 76 | We're here you're not here | Tongjiang county | chant | hypertonic | unknown | 2:30 |
| 77 | Spring arrives at Longmen mountain | The whole Sichuan province | voiceless sound | lyric | unknown | 6:06 |
| 78 | Shuyun fengliu | The whole Sichuan province | voiceless sound | lyric | unknown | 2:53 |
| 79 | Little kite flying | The whole Sichuan province | voiceless sound | lyric | unknown | 2:44 |
| 80 | The sun rises with joy | Eastern Sichuan | minore | cheerful | Jin Gu | 1:33 |
| 81 | Lialing river tune | Jialing zone | folk song | hypertonic | Huang Huwei | 7:03 |
| 82 | Herding cows | Da county | folk song | hypertonic | Dan Zhaoyi | 3:11 |
| 83 | Morning song | Pujiang county | folk song | lyric | Huang Huwei | 1:05 |
| 84 | Lyric ballad | Jiangyou county | folk song | lyric | Huang Huwei | 1:30 |
| 85 | Spring suburbs of rongcheng | Chengdu city | folk song | cheerful | Huang Huwei | 3:15 |
| 86 | Aba night club | Mao county | folk song | cheerful | Huang Huwei | 5:39 |

**Table S2.** All the 1548 *rHR* values in systematic subject-to-sample response (SSTSR) profile

| Music sample | Testee subject | | | | | | | | | | | | | | | | | | *mean* | *s.e.* |
|---|---|---|---|---|---|---|---|---|---|---|---|---|---|---|---|---|---|---|---|---|
| | P1 | P2 | P3 | P4 | P5 | P6 | P7 | P8 | P9 | P10 | P11 | P12 | P13 | P14 | P15 | P16 | P17 | P18 | | |
| Willow and willow | 0.094 | 0.138 | 0.132 | 0.050 | 0.054 | 0.018 | 0.115 | 0.059 | 0.041 | 0.058 | 0.024 | 0.098 | 0.063 | 0.116 | -0.017 | 0.040 | 0.094 | 0.147 | 0.074 | 0.044 |
| Ten flowers blooming | 0.113 | 0.094 | 0.066 | 0.023 | 0.018 | 0.079 | 0.033 | 0.019 | 0.127 | 0.040 | 0.072 | 0.072 | 0.058 | 0.071 | 0.077 | -0.013 | 0.061 | 0.190 | 0.067 | 0.045 |
| Grape picking | 0.041 | 0.146 | 0.015 | 0.065 | 0.099 | 0.040 | 0.059 | 0.051 | 0.075 | 0.074 | 0.088 | 0.054 | 0.052 | 0.134 | 0.119 | 0.013 | 0.076 | 0.143 | 0.075 | 0.039 |
| Boy whistles to cross the ditch | 0.087 | 0.018 | 0.141 | 0.044 | 0.190 | 0.099 | 0.102 | 0.157 | 0.050 | 0.029 | 0.055 | 0.134 | 0.142 | 0.044 | 0.051 | 0.159 | 0.001 | 0.052 | 0.086 | 0.054 |
| Pouch embroidering | 0.030 | 0.101 | 0.094 | 0.162 | -0.003 | 0.036 | 0.094 | 0.078 | 0.065 | 0.102 | 0.039 | 0.057 | 0.060 | 0.040 | 0.183 | 0.069 | 0.149 | 0.108 | 0.081 | 0.047 |
| When will the pagoda tree blossom | 0.025 | -0.038 | 0.056 | 0.089 | 0.072 | 0.101 | 0.152 | 0.107 | 0.148 | 0.141 | 0.119 | 0.122 | 0.073 | 0.033 | 0.102 | 0.039 | -0.006 | 0.046 | 0.077 | 0.052 |
| Mianzhu love song | 0.099 | 0.024 | 0.083 | 0.110 | 0.081 | 0.029 | 0.079 | 0.076 | 0.138 | 0.068 | 0.060 | 0.045 | 0.110 | 0.070 | 0.058 | 0.086 | 0.118 | 0.051 | 0.077 | 0.030 |
| I came up the mountain with my mover | -0.055 | 0.007 | 0.101 | 0.114 | 0.111 | 0.112 | 0.017 | 0.124 | 0.070 | 0.100 | 0.026 | 0.076 | 0.158 | 0.078 | 0.082 | 0.038 | 0.028 | -0.024 | 0.065 | 0.055 |
| See lover off song | 0.182 | 0.004 | 0.143 | 0.041 | 0.107 | 0.136 | 0.088 | -0.001 | 0.041 | 0.118 | 0.063 | 0.233 | 0.100 | 0.099 | 0.138 | 0.033 | -0.038 | 0.027 | 0.084 | 0.067 |
| Nanmu carrying pole shaking | 0.112 | 0.044 | 0.085 | 0.094 | 0.089 | 0.101 | 0.115 | 0.143 | 0.031 | 0.132 | 0.088 | 0.049 | 0.050 | 0.113 | 0.115 | 0.094 | 0.143 | 0.006 | 0.089 | 0.038 |
| Climbing peas | 0.088 | 0.054 | 0.031 | 0.031 | 0.106 | 0.031 | 0.147 | 0.073 | -0.005 | 0.157 | 0.033 | 0.189 | 0.085 | 0.113 | 0.155 | 0.116 | 0.076 | 0.174 | 0.092 | 0.055 |
| Airing clothes | 0.028 | 0.045 | 0.121 | 0.097 | 0.137 | 0.118 | 0.072 | 0.028 | 0.121 | 0.035 | 0.037 | 0.151 | 0.137 | 0.061 | 0.043 | 0.095 | 0.096 | 0.117 | 0.086 | 0.041 |
| Mowing song | 0.165 | 0.049 | 0.163 | 0.053 | 0.093 | 0.094 | 0.106 | 0.109 | 0.123 | 0.145 | 0.122 | 0.106 | 0.091 | 0.010 | 0.123 | 0.118 | 0.045 | 0.067 | 0.099 | 0.040 |
| A diligent and cheerful Person loved by a girl | 0.138 | 0.117 | 0.080 | 0.024 | 0.118 | 0.078 | 0.094 | 0.114 | 0.146 | 0.092 | 0.079 | 0.102 | 0.112 | 0.015 | -0.006 | 0.080 | 0.109 | 0.032 | 0.085 | 0.042 |
| Follow the sun all the way | 0.026 | 0.048 | 0.116 | 0.096 | 0.171 | 0.033 | 0.160 | 0.076 | 0.094 | 0.094 | 0.137 | 0.106 | 0.074 | 0.121 | 0.082 | 0.147 | 0.092 | 0.012 | 0.094 | 0.044 |
| The sun is shining overhead | 0.077 | 0.069 | 0.077 | 0.123 | 0.085 | 0.042 | 0.010 | 0.075 | 0.000 | 0.077 | 0.087 | 0.100 | 0.159 | 0.045 | 0.070 | 0.072 | 0.033 | -0.038 | 0.065 | 0.044 |
| The sun sets in Xiya | 0.019 | 0.103 | 0.170 | 0.032 | 0.105 | 0.004 | 0.067 | 0.159 | 0.115 | 0.069 | 0.103 | 0.118 | 0.108 | 0.084 | 0.070 | 0.169 | 0.085 | 0.129 | 0.095 | 0.046 |
| Tell me to sing, I won't push | 0.043 | 0.034 | 0.095 | 0.144 | 0.123 | 0.041 | 0.103 | 0.066 | 0.043 | 0.060 | 0.117 | 0.065 | 0.102 | 0.045 | -0.020 | 0.052 | 0.067 | 0.062 | 0.069 | 0.038 |
| The moon slants westward | 0.050 | 0.167 | 0.112 | 0.077 | 0.066 | 0.158 | 0.083 | 0.088 | 0.121 | 0.171 | 0.090 | 0.060 | 0.026 | 0.063 | 0.054 | 0.046 | 0.047 | 0.093 | 0.087 | 0.042 |
| The small boat is empty at both ends | 0.139 | 0.114 | 0.128 | 0.118 | 0.067 | 0.084 | 0.152 | 0.040 | 0.061 | 0.093 | 0.052 | 0.152 | 0.102 | 0.150 | 0.055 | 0.190 | -0.002 | 0.202 | 0.105 | 0.053 |
| The moon sets in the west | 0.199 | 0.151 | -0.010 | 0.100 | 0.019 | 0.069 | 0.113 | -0.058 | 0.062 | 0.073 | 0.060 | 0.169 | 0.126 | 0.016 | 0.147 | -0.011 | 0.119 | 0.025 | 0.076 | 0.068 |
| Ten fan tuning | 0.115 | 0.060 | 0.009 | 0.000 | 0.073 | 0.061 | 0.038 | 0.079 | 0.029 | 0.061 | 0.107 | 0.148 | 0.087 | 0.148 | 0.111 | 0.051 | 0.134 | 0.092 | 0.078 | 0.043 |
| Kangding love song | 0.090 | 0.017 | 0.184 | 0.126 | 0.080 | 0.104 | -0.037 | 0.140 | -0.042 | 0.148 | 0.029 | 0.000 | 0.099 | 0.087 | 0.108 | 0.164 | 0.067 | 0.096 | 0.081 | 0.063 |

| | | | | | | | | | | | | | | | | | | | | |
|---|---|---|---|---|---|---|---|---|---|---|---|---|---|---|---|---|---|---|---|---|
| The snow on the mountain is like a flower | 0.195 | 0.107 | 0.069 | 0.052 | 0.067 | 0.129 | 0.080 | 0.076 | 0.095 | 0.084 | 0.134 | 0.139 | 0.030 | 0.086 | 0.089 | 0.124 | 0.072 | 0.109 | 0.097 | 0.037 |
| Cows in the land of fortune | -0.065 | -0.015 | 0.180 | 0.088 | 0.065 | 0.109 | 0.120 | 0.179 | 0.093 | 0.222 | 0.092 | 0.091 | 0.088 | 0.031 | 0.075 | 0.172 | 0.119 | 0.127 | 0.098 | 0.067 |
| My hometown is by the Yalong river | 0.098 | 0.174 | 0.109 | 0.147 | 0.085 | 0.146 | 0.083 | 0.081 | 0.098 | 0.181 | 0.086 | 0.155 | 0.071 | 0.077 | 0.123 | 0.122 | 0.090 | 0.113 | 0.113 | 0.033 |
| You are the red coral | 0.150 | 0.086 | 0.096 | 0.042 | -0.009 | 0.086 | 0.072 | 0.200 | 0.086 | 0.084 | 0.123 | 0.134 | 0.050 | 0.215 | 0.008 | 0.108 | 0.142 | 0.019 | 0.094 | 0.059 |
| Please lend me your wings | 0.033 | 0.095 | 0.112 | 0.079 | 0.046 | 0.139 | 0.055 | 0.137 | 0.034 | 0.045 | 0.124 | 0.063 | 0.056 | 0.056 | 0.092 | 0.061 | 0.076 | 0.091 | 0.077 | 0.033 |
| I'm familiar with it, it's my own brother | -0.026 | 0.071 | 0.155 | 0.148 | 0.048 | 0.120 | -0.047 | 0.057 | 0.053 | 0.056 | 0.037 | 0.186 | 0.111 | 0.127 | 0.002 | 0.064 | 0.082 | 0.031 | 0.071 | 0.060 |
| Where does Guozhuang Jump from | 0.073 | 0.052 | 0.053 | -0.007 | 0.072 | 0.117 | 0.044 | 0.126 | 0.079 | 0.047 | 0.076 | 0.016 | 0.054 | 0.020 | 0.004 | 0.101 | 0.126 | 0.079 | 0.063 | 0.038 |
| Meet together | 0.050 | 0.106 | 0.177 | 0.149 | 0.099 | 0.122 | 0.101 | 0.076 | 0.092 | 0.089 | 0.155 | -0.011 | 0.108 | 0.057 | 0.072 | 0.085 | 0.080 | 0.090 | 0.094 | 0.041 |
| Life used to be very difficult | 0.103 | 0.081 | 0.055 | 0.113 | 0.015 | 0.050 | 0.024 | 0.057 | 0.119 | 0.047 | 0.169 | 0.133 | 0.120 | 0.128 | 0.042 | 0.080 | 0.065 | 0.084 | 0.083 | 0.041 |
| Cannot be divided | 0.051 | 0.059 | 0.056 | 0.140 | 0.092 | 0.069 | 0.020 | 0.086 | 0.075 | 0.071 | 0.112 | 0.089 | 0.126 | 0.060 | 0.127 | 0.138 | 0.109 | 0.083 | 0.087 | 0.032 |
| Where is the girl? Where am I | 0.135 | 0.164 | 0.017 | 0.028 | 0.051 | 0.036 | 0.092 | 0.220 | 0.031 | 0.005 | 0.026 | 0.093 | 0.044 | 0.100 | 0.081 | 0.159 | 0.122 | 0.065 | 0.082 | 0.058 |
| Picking pears | 0.094 | 0.007 | 0.166 | 0.053 | 0.174 | 0.126 | 0.051 | 0.087 | 0.050 | 0.086 | 0.030 | 0.032 | 0.093 | 0.092 | 0.060 | 0.028 | 0.078 | 0.054 | 0.076 | 0.044 |
| Green and lush mountains | 0.178 | 0.104 | 0.065 | 0.130 | -0.009 | 0.027 | 0.115 | 0.183 | 0.207 | 0.121 | 0.100 | 0.054 | -0.007 | 0.035 | 0.050 | 0.003 | 0.071 | 0.094 | 0.085 | 0.063 |
| Hi, naginara | 0.146 | 0.094 | 0.107 | 0.089 | 0.162 | 0.079 | 0.129 | 0.041 | 0.088 | 0.007 | 0.066 | 0.071 | 0.119 | 0.161 | 0.015 | 0.071 | 0.052 | 0.058 | 0.086 | 0.044 |
| Narra | 0.004 | 0.101 | 0.099 | 0.066 | 0.118 | 0.068 | 0.148 | 0.069 | 0.024 | 0.151 | 0.140 | 0.087 | 0.023 | 0.031 | 0.151 | 0.089 | 0.164 | 0.064 | 0.089 | 0.048 |
| That nagging | 0.127 | 0.097 | 0.140 | 0.180 | 0.118 | 0.015 | 0.000 | 0.101 | 0.097 | 0.086 | 0.106 | 0.058 | 0.065 | 0.140 | -0.022 | 0.139 | 0.081 | 0.115 | 0.091 | 0.051 |
| Tiaoxi minore | 0.088 | 0.096 | 0.058 | 0.048 | 0.177 | 0.180 | 0.037 | 0.130 | 0.097 | 0.075 | 0.004 | 0.055 | 0.074 | 0.029 | 0.111 | 0.019 | 0.127 | 0.049 | 0.081 | 0.049 |
| Picking flowers | 0.072 | 0.136 | 0.087 | 0.156 | 0.114 | 0.126 | 0.131 | 0.106 | 0.026 | 0.152 | 0.001 | 0.065 | 0.059 | 0.051 | 0.058 | 0.126 | 0.119 | 0.053 | 0.091 | 0.044 |
| Make a fuss at five o'clock | -0.001 | 0.109 | 0.087 | 0.066 | 0.199 | 0.115 | -0.041 | 0.105 | 0.092 | 0.105 | 0.104 | 0.086 | 0.085 | 0.117 | 0.151 | 0.042 | 0.038 | 0.138 | 0.089 | 0.053 |
| Enjoy the lantern festival | 0.002 | 0.060 | 0.024 | 0.035 | 0.001 | 0.103 | 0.120 | 0.087 | -0.020 | 0.000 | 0.097 | 0.067 | 0.054 | 0.130 | 0.151 | 0.126 | 0.093 | 0.062 | 0.066 | 0.050 |
| A military song | -0.045 | 0.068 | 0.068 | 0.117 | 0.041 | 0.071 | 0.100 | 0.094 | 0.089 | 0.075 | -0.072 | 0.082 | 0.097 | 0.150 | 0.050 | 0.060 | -0.010 | 0.103 | 0.063 | 0.054 |
| Military song | 0.156 | 0.068 | 0.173 | 0.079 | 0.109 | 0.103 | -0.018 | 0.075 | 0.144 | 0.148 | 0.075 | 0.134 | 0.086 | 0.038 | 0.069 | 0.159 | 0.070 | 0.118 | 0.099 | 0.047 |
| Persuade new sister | 0.146 | 0.026 | 0.046 | 0.171 | 0.156 | 0.087 | 0.089 | 0.147 | 0.099 | 0.040 | 0.076 | 0.069 | 0.056 | 0.113 | 0.126 | 0.089 | 0.063 | 0.129 | 0.096 | 0.042 |
| Counting flowers | 0.114 | 0.100 | 0.082 | 0.089 | 0.061 | 0.108 | 0.194 | 0.090 | 0.039 | 0.073 | 0.155 | 0.034 | 0.058 | 0.128 | 0.143 | 0.062 | 0.075 | 0.115 | 0.096 | 0.040 |
| To flower | 0.025 | 0.111 | 0.095 | 0.068 | 0.086 | 0.059 | 0.136 | 0.151 | 0.080 | 0.091 | 0.149 | 0.154 | 0.085 | 0.120 | 0.015 | 0.112 | 0.098 | 0.152 | 0.099 | 0.040 |
| Embroidered pillow | -0.056 | -0.035 | 0.049 | -0.034 | 0.101 | 0.062 | 0.124 | 0.164 | 0.109 | 0.114 | 0.115 | 0.002 | 0.087 | 0.161 | 0.085 | 0.046 | -0.007 | -0.016 | 0.060 | 0.067 |

| | | | | | | | | | | | | | | | | | | | | |
|---|---|---|---|---|---|---|---|---|---|---|---|---|---|---|---|---|---|---|---|---|
| Pairing song | 0.107 | 0.158 | 0.046 | 0.140 | 0.079 | 0.140 | 0.089 | 0.173 | -0.049 | 0.008 | 0.072 | 0.071 | 0.137 | -0.044 | 0.002 | 0.081 | 0.067 | 0.082 | 0.076 | 0.063 |
| Pruning plum blossoms | 0.069 | -0.014 | 0.133 | 0.113 | 0.054 | 0.033 | 0.142 | 0.115 | 0.041 | 0.066 | 0.055 | 0.152 | 0.094 | 0.092 | 0.107 | 0.000 | 0.080 | 0.108 | 0.080 | 0.045 |
| Cursing matchmakers | 0.120 | 0.058 | 0.132 | 0.072 | 0.102 | 0.096 | 0.090 | 0.078 | 0.183 | 0.095 | 0.045 | 0.028 | 0.092 | 0.111 | 0.070 | 0.010 | 0.126 | 0.086 | 0.089 | 0.039 |
| Carrying a thousand pounds across Ba mountain | 0.092 | 0.106 | 0.074 | 0.086 | 0.041 | 0.133 | 0.115 | 0.135 | 0.116 | 0.035 | 0.063 | 0.014 | 0.100 | 0.061 | 0.059 | 0.012 | -0.008 | 0.019 | 0.070 | 0.043 |
| Heavy back makes it difficult to climb the slope | 0.105 | 0.053 | 0.107 | 0.097 | 0.014 | 0.076 | 0.075 | 0.031 | 0.079 | 0.128 | 0.139 | 0.086 | 0.105 | 0.001 | 0.082 | 0.090 | 0.117 | 0.128 | 0.084 | 0.038 |
| It's difficult to climb uphill with your back on your back | 0.082 | 0.022 | 0.072 | 0.054 | 0.049 | 0.151 | 0.092 | 0.141 | 0.120 | 0.063 | 0.193 | 0.020 | 0.090 | 0.114 | 0.138 | 0.066 | -0.009 | 0.036 | 0.083 | 0.051 |
| A cool breeze is coming | 0.113 | 0.107 | 0.041 | 0.045 | 0.044 | 0.003 | 0.077 | 0.102 | 0.080 | 0.162 | 0.098 | 0.031 | 0.065 | 0.131 | 0.029 | 0.085 | 0.022 | 0.096 | 0.074 | 0.041 |
| Pairs of rice paddies for field harvesting | 0.039 | 0.063 | 0.101 | 0.114 | 0.175 | 0.057 | 0.104 | 0.067 | 0.065 | 0.072 | 0.168 | 0.099 | 0.145 | 0.055 | -0.012 | 0.149 | 0.066 | 0.137 | 0.092 | 0.048 |
| Lazy turnover | 0.086 | 0.089 | 0.113 | 0.075 | 0.127 | -0.005 | 0.108 | 0.076 | 0.115 | 0.032 | 0.015 | 0.125 | -0.018 | 0.061 | -0.018 | 0.098 | 0.204 | 0.159 | 0.080 | 0.059 |
| Happy life, sweet and savory | 0.051 | 0.103 | 0.071 | 0.071 | 0.086 | 0.130 | 0.022 | 0.116 | 0.066 | 0.065 | 0.079 | 0.126 | 0.002 | 0.062 | 0.152 | 0.100 | 0.040 | 0.141 | 0.082 | 0.040 |
| Bean sprouts, sallion and garlic leaves | 0.107 | 0.078 | 0.101 | 0.069 | 0.080 | 0.053 | 0.196 | 0.013 | -0.040 | 0.079 | 0.159 | 0.189 | -0.023 | 0.089 | 0.101 | 0.091 | 0.070 | 0.090 | 0.083 | 0.060 |
| Chasing wild cats | 0.090 | -0.029 | 0.038 | 0.111 | 0.098 | 0.079 | -0.019 | 0.085 | 0.074 | 0.110 | 0.189 | 0.099 | 0.009 | 0.077 | 0.097 | 0.083 | 0.070 | 0.080 | 0.075 | 0.049 |
| Emei restaurant | 0.051 | 0.115 | 0.133 | 0.042 | 0.056 | 0.142 | 0.040 | 0.063 | 0.098 | 0.112 | 0.065 | 0.109 | 0.157 | 0.052 | 0.042 | -0.014 | 0.031 | 0.069 | 0.076 | 0.044 |
| I haven't been here for a long time | 0.105 | 0.115 | 0.087 | 0.166 | 0.096 | 0.047 | 0.091 | 0.020 | 0.086 | 0.021 | 0.060 | 0.085 | 0.062 | 0.011 | 0.120 | 0.130 | 0.091 | 0.091 | 0.082 | 0.039 |
| Froggy | 0.049 | 0.132 | 0.088 | 0.172 | 0.142 | 0.106 | 0.111 | 0.105 | 0.124 | 0.078 | 0.119 | 0.225 | -0.010 | 0.016 | 0.069 | 0.108 | 0.033 | 0.104 | 0.098 | 0.054 |
| Cattle herding song | 0.077 | 0.066 | 0.061 | 0.072 | 0.053 | 0.133 | 0.063 | 0.133 | 0.072 | 0.083 | -0.023 | 0.086 | 0.076 | 0.038 | 0.233 | 0.130 | 0.001 | 0.003 | 0.075 | 0.056 |
| Shepherd song | 0.132 | 0.155 | 0.034 | -0.038 | 0.167 | 0.068 | 0.095 | 0.054 | 0.079 | 0.057 | 0.086 | 0.060 | 0.194 | 0.109 | 0.086 | 0.072 | 0.059 | 0.062 | 0.085 | 0.052 |
| Pony running | 0.061 | 0.073 | -0.002 | 0.085 | 0.107 | 0.052 | 0.015 | 0.110 | 0.163 | 0.161 | 0.152 | 0.027 | 0.177 | 0.049 | 0.222 | 0.061 | 0.045 | 0.109 | 0.093 | 0.060 |
| Step by step tight | 0.111 | 0.020 | 0.103 | 0.098 | 0.012 | 0.098 | 0.147 | 0.086 | 0.114 | 0.066 | 0.069 | 0.105 | 0.142 | 0.046 | 0.112 | 0.103 | 0.037 | 0.069 | 0.085 | 0.037 |
| Three sharp sounds | 0.060 | 0.103 | 0.178 | 0.120 | 0.088 | 0.076 | 0.115 | 0.034 | 0.031 | 0.060 | 0.118 | 0.119 | -0.046 | 0.064 | 0.073 | -0.021 | 0.118 | 0.199 | 0.083 | 0.059 |
| Top-ten generals | 0.083 | 0.161 | 0.051 | 0.104 | 0.115 | 0.049 | 0.029 | 0.142 | 0.162 | 0.124 | 0.051 | 0.085 | 0.068 | 0.047 | 0.119 | 0.131 | 0.103 | 0.039 | 0.092 | 0.041 |
| December visit | 0.092 | 0.120 | 0.028 | 0.103 | 0.024 | 0.000 | -0.048 | 0.081 | 0.120 | 0.105 | 0.063 | 0.113 | 0.053 | 0.006 | 0.040 | 0.114 | 0.105 | 0.061 | 0.066 | 0.048 |
| New Year comes in January | 0.164 | 0.152 | 0.056 | 0.088 | 0.134 | 0.106 | 0.031 | 0.119 | 0.075 | 0.072 | 0.103 | 0.137 | 0.058 | 0.138 | 0.023 | 0.107 | 0.041 | 0.086 | 0.094 | 0.041 |
| Elegiac song | 0.110 | 0.127 | 0.046 | -0.012 | 0.073 | 0.123 | 0.130 | 0.064 | 0.047 | 0.139 | 0.100 | 0.110 | 0.165 | 0.142 | 0.060 | 0.078 | 0.035 | 0.012 | 0.086 | 0.048 |
| Pinglu chant | 0.168 | 0.127 | 0.055 | 0.151 | 0.013 | 0.026 | 0.122 | 0.139 | 0.058 | -0.008 | 0.073 | 0.130 | 0.106 | 0.034 | 0.069 | 0.013 | 0.024 | 0.067 | 0.076 | 0.052 |
| Miss Yao is very different | 0.108 | 0.077 | 0.090 | 0.058 | 0.087 | 0.145 | 0.102 | 0.017 | 0.008 | 0.156 | 0.089 | 0.167 | 0.112 | 0.098 | 0.069 | 0.033 | -0.011 | 0.016 | 0.079 | 0.050 |

| | | | | | | | | | | | | | | | | | | | | |
|---|---|---|---|---|---|---|---|---|---|---|---|---|---|---|---|---|---|---|---|---|
| We're here you're not here | 0.038 | 0.103 | 0.070 | 0.055 | 0.122 | 0.128 | 0.136 | 0.096 | 0.056 | 0.108 | 0.063 | 0.099 | 0.093 | 0.059 | 0.055 | -0.034 | 0.108 | -0.013 | 0.075 | 0.044 |
| Spring arrives at Longmen mountain | 0.126 | 0.124 | 0.079 | 0.029 | 0.164 | 0.096 | 0.057 | 0.070 | -0.010 | 0.058 | 0.116 | 0.069 | 0.051 | 0.106 | 0.133 | 0.118 | 0.069 | 0.041 | 0.083 | 0.042 |
| Shuyun fengliu | -0.017 | 0.068 | 0.117 | 0.015 | 0.078 | 0.132 | 0.053 | 0.066 | 0.155 | 0.061 | 0.164 | -0.047 | 0.007 | 0.005 | 0.010 | 0.094 | 0.173 | 0.021 | 0.064 | 0.063 |
| Little kite flying | 0.078 | 0.082 | 0.052 | 0.005 | 0.130 | 0.066 | 0.086 | 0.066 | 0.090 | 0.016 | 0.068 | 0.015 | 0.128 | 0.105 | 0.112 | 0.090 | 0.096 | 0.044 | 0.074 | 0.036 |
| The sun rises with joy | 0.046 | 0.087 | 0.063 | 0.027 | 0.161 | 0.054 | 0.049 | 0.070 | 0.131 | 0.097 | 0.145 | 0.074 | 0.073 | 0.107 | 0.114 | 0.108 | 0.006 | 0.014 | 0.079 | 0.042 |
| Lialing river tune | 0.145 | 0.033 | 0.175 | 0.094 | 0.039 | 0.042 | 0.049 | 0.117 | 0.012 | 0.102 | 0.101 | 0.060 | 0.084 | 0.082 | 0.053 | 0.147 | 0.010 | 0.059 | 0.078 | 0.046 |
| Herding cows | 0.113 | 0.087 | 0.096 | 0.095 | 0.023 | 0.039 | 0.093 | 0.043 | 0.055 | 0.083 | 0.086 | 0.106 | 0.056 | 0.054 | 0.079 | 0.038 | 0.133 | 0.066 | 0.075 | 0.029 |
| Morning song | 0.032 | 0.100 | 0.104 | 0.029 | 0.049 | 0.070 | 0.121 | 0.013 | 0.114 | 0.071 | 0.044 | 0.106 | 0.065 | -0.019 | 0.028 | 0.160 | 0.161 | 0.115 | 0.076 | 0.049 |
| Lyric ballad | 0.089 | 0.035 | 0.059 | 0.069 | 0.137 | 0.058 | 0.053 | 0.023 | -0.009 | 0.062 | 0.075 | -0.029 | 0.085 | 0.099 | 0.046 | 0.035 | 0.046 | 0.088 | 0.057 | 0.038 |
| Spring suburbs of rongcheng | 0.145 | 0.083 | 0.068 | 0.132 | 0.139 | 0.118 | 0.176 | 0.043 | 0.170 | -0.057 | 0.024 | 0.008 | 0.039 | 0.073 | 0.157 | 0.078 | 0.109 | 0.101 | 0.089 | 0.060 |
| Aba night club | 0.072 | 0.013 | 0.030 | 0.142 | 0.053 | 0.064 | 0.111 | 0.069 | 0.098 | 0.175 | 0.096 | 0.046 | 0.174 | 0.099 | 0.087 | 0.030 | 0.112 | 0.022 | 0.083 | 0.047 |
